# Incidence of Acute and Chronic Post-Thoracotomy Pain in Pediatric Patients

**DOI:** 10.3390/children8080642

**Published:** 2021-07-27

**Authors:** Giuliano Marchetti, Alessandro Vittori, Fabio Ferrari, Elisa Francia, Ilaria Mascilini, Emiliano Petrucci, Simone Piga, Valerio Pardi, Marco Cascella, Giorgia Contini, Franco Marinangeli, Alessandro Inserra, Sergio Giuseppe Picardo

**Affiliations:** 1 Department of Anesthesia and Critical Care, ARCO Roma, Ospedale Pediatrico Bambino Gesù, IRCCS, Piazza S. Onofrio 4, 00165 Rome, Italy; alexvittori@libero.it (A.V.); fabio.ferrari@opbg.net (F.F.); elisa.francia@opbg.net (E.F.); ilaria.mascilini@opbg.net (I.M.); sgiuseppe.picardo@opbg.net (S.G.P.); 2 Department of Anesthesia and Intensive Care Unit, San Salvatore Academic Hospital of L’Aquila, Via Vetoio, 48, 67100 L’Aquila, Italy; petrucciemiliano@gmail.com; 3 Unit of Clinical Epidemiology, Ospedale Pediatrico Bambino Gesù, IRCCS, Piazza S. Onofrio 4, 00165 Rome, Italy; simone.piga@opbg.net; 4 Surgical Department, General and Thoracic Unit, Ospedale Pediatrico Bambino Gesù, IRCCS, Piazza S. Onofrio 4, 00165 Rome, Italy; valerio.pardi@opbg.net (V.P.); giorgia.contini@opbg.net (G.C.); alessandro.inserra@opbg.net (A.I.); 5 Department of Anesthesia and Critical Care, Istituto Nazionale Tumori-IRCCS, Fondazione Pascale, Via Mariano Semmola, 53, 80131 Naples, Italy; m.cascella@istitutotumori.na.it; 6 Department of Anesthesiology, Intensive Care and Pain Treatment, University of L’Aquila, Piazzale Salvatore Tommasi, 1, 67100 Coppito, Italy; francomarinangeli@gmail.com

**Keywords:** pain, thoracic surgery, anesthesia, children, pediatric anesthesia, chronic pain, acute pain, pain management index, neuropathic pain, chronic post-thoracotomy pain syndrome

## Abstract

We studied acute and chronic pain in pediatric patients who underwent thoracotomy for benign disease with a follow-up of at least three months. A telephone interview investigated about the presence of pain and the analgesic therapy in progress. The results were compared with the anesthetic technique, postoperative pain and the adequacy of pain therapy, both during the first week after surgery and at the time of interview. Fifty-six families consented to the study. The mean age of the children at surgery was 2.9 ± 4.5 years, while at the time of the interview was 6.5 ± 4.4 years. We performed different anesthetic strategies: Group A: general anesthesia (36 pts); Group B: general anesthesia and thoracic epidural (10 pts); Group C: general anesthesia and intercostal nerve block (10 pts). During the immediate postoperative period, 21 patients (37.5%) had at least one painful episode. At the time of interview, 3 children (5.3%) had moderate chronic neuropathic (burning) pain on surgical scar. There was no statistically significant difference between the type of anesthesia and the incidence and severity of acute post-operative pain. Despite its limitations, this study confirms the low incidence of chronic post-thoracotomy pain syndrome in children.

## 1. Introduction

Thoracotomy is one of the most painful surgical procedures, not only in the immediate postoperative period, but also in the longer term after surgery [1]. Despite the improvements in perioperative care, acute and chronic pain after thoracotomy still remains a widespread issue [2]. In adults, the incidence of chronic post-thoracotomy pain syndrome (CPTPS) varies from 26% to 91% [3,4,5,6,7,8,9]. In particular, up to 60% of patients reported pain one month after surgery, while 35–50% reported pain after one or two years [3,4]. The etiology of CPTPS is poorly understood, although it seems to be linked to the combination of nociceptive pain, neuropathic pain, nerve damage, central sensitization and tumor recurrence [5].

In children, postoperative pain is often underestimated or misunderstood, both during hospitalization and, above all, at home [6,10]. The few studies on the prevalence of CPTPS in the pediatric age showed that a lower risk of developing chronic pain exists. Kristensen et al. [7] described that 16% of 88 adult patients who underwent thoracotomy in childhood or youth recalled their pain lasted more than 3 months, while 3.4% still had pain at the time of the study. Furthermore, Chou et al. found that only one patient out of 51 children (1.94%) operated for non-elective thoracotomy [8] had chronic pain at the time of the interview. In addition, surveys on patients who have undergone an amputation or hernia have suggested that the young age at the time of surgery is associated with a lower risk of developing chronic pain [5,9]. The mechanisms of this apparent protection to the development of chronic pain in children are unknown [11].

This study was aimed at evaluating the incidence of CPTPS in pediatric patients aged between 0 and 18 years old and at least 3 months after surgery.

## 2. Materials and Methods

### 2.1. Study Methodology

**Figure 1:**
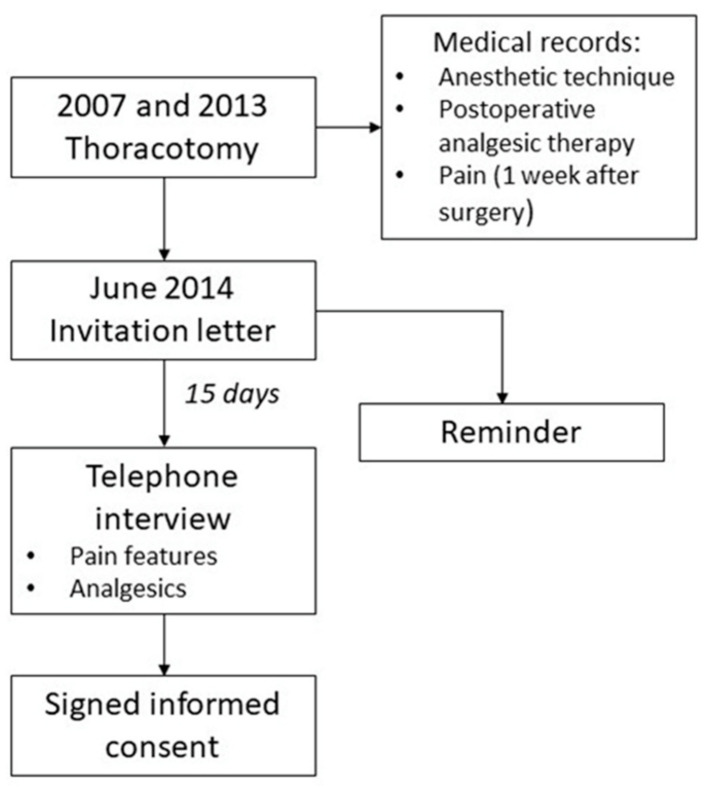
Flow-chart of the study.

This retrospective cross-sectional study has been approved by the Ethics Committee of Ospedale Pediatrico Bambino Gesù IRCCS on 06/11/2013 with number 957/RA and was articulated in the following way:

(1) The study included patients who underwent thoracotomy between 2007 and 2013 for benign diseases; children underwent a follow-up of at least three months and did not reach the age of majority (over 18 years old) in November 2013.
(2) In June 2014, the families received a letter explaining the purpose and the method of the study and the request to collaborate. To prepare parents for the telephone interview, together with the letter a questionnaire for data collection, an informed consent was sent.
(3) Fifteen days after the letters were sent, the telephone interview was performed. After establishing a positive contact, we asked them to return us via email, fax, or post the signed informed consent.
(4) During the interview, we asked about the presence, type, intensity and location of the pain at the time of the interview and any analgesic therapy in progress.
(5) Using the medical records referring to the period of hospitalization for surgery, we investigated the anesthetic technique used, the postoperative analgesic therapy prescribed and changes in pain during the first week of the postoperative period.
(6) In the case of unsuccessful telephone contact, a second letter was sent as a reminder (Figure 1).

### 2.2. Exclusion Criteria

Patients who reached the age of majority in November 2013, patients with malignant and inflammatory diseases, patients who did not have a follow-up of at least three months and patients of whom it was not possible to have an answer after the second letter of presentation were excluded from the study. Subjects removed from the study in case of non-response to the second letter, were not replaced.

### 2.3. Pain Assessment

The prevalence of acute postoperative pain and the prevalence of chronic pain at the time of interview was investigated. The adequacy of pain treatment was assessed through the Pain Management Index (PMI) modified for the use on children [12]. It compares the analgesic score (no analgesic = 0 points; WHO I = 1 point; WHO II = 2 points; WHO III = 3 points) with pain score using the numeric rate scale (NRS), the Face, Legs, Activity, Cry, Consolability (FLACC) scale and Crying Requires oxygen Increased vital signs Expression Sleep (CRIES) scale. In particular, NRS, FLACC and CRIES scores of 0 indicate no pain and receive 0 points; scores of 1–3 indicate mild pain and receive 1 point; scores of 4–6 indicate moderate pain and receive 2 points; scores of 7–10 indicate severe pain and receive 3 points. The CRIES scale it has been used for infants over 32 weeks of age, the FLACC scale for children over 30 days of age and under 4 years of age, while the NRS scales for patients over 4 years of age.

Moreover, with regard to the Neonatal Infant Pain Profile scale (NIPS) used for premature neonates under 32 weeks of age, scores of 0–2 indicate no pain and receive 0 points; scores of 3–4 indicate mild pain and receive 1 point; scores of 5–7 indicate severe pain and receive 3 points.

Finally, the COMFORT scale provides a pain rating between 9 and 45 based on nine different parameters, each rated from 1 to 5. Scores of 9 indicates no pain and receive 0 points; 9–17 indicate no pain and receive 0 points; 18–26 indicate mild pain and received 1 point; 27–35 indicate moderate pain and receive 2 points; finally, scores of 36–45 indicate severe pain and receive 3 points. The COMFORT scale was used for sedated patients.

PMI was computed by subtracting pain score from analgesic score. It ranges from −3 (patient with severe pain receiving no drug) to +3 (patient receiving strong opioids and reporting no pain); negative scores indicate undertreatment.

The anesthetic technique used at the time of surgery was evaluated. Furthermore, postoperative pain and the adequacy of pain therapy, both during the first week after surgery and at the time of interview were evaluated [13,14,15]. Postoperative pain and the efficacy of the administered therapy were assessed using the hospital protocol. It suggests the use of a several pain assessment scale based on the patient’s age, the intensity of postoperative care required by the patients’ clinical condition upon leaving the operating room. Moreover, the protocol provides that pain assessment is recommended upon returning to the ward after surgery and every eight hours there-after or whenever necessary

### 2.4. Statistical Analysis

Categorical variables were summarized using absolute frequencies and percentages and continuous variables by the mean or median, as appropriate. To determine statistical differences between groups, we used the Chi-square test or Fischer’s exact test for categorical variables and the t-test or Mann–Whitney test for continuous variables.

We performed comparisons of the three treatment groups with gender and median age in years (category with values equal to or greater than the median and category with values below the median) at surgery and treatment groups with median age in years (category with values equal to or greater than the median and category with values below the median) at interview. The results of the Chi-square test are not statistically significant.

The sample size of 56 patients allows us to estimate the proportion of pain with an error of 13.1%, considering an alpha level = 0.05 and a confidence level of 95%. In this study, based on the estimate of the proportion of pain, we can rely on the measure of error of the estimate which is 13.1% considering a maximum variability (50%) which provides the most conservative estimate. The power of the study in this case cannot be detected as we do not have an effect size. Statistical analyses were carried out using the Stata program, version 13 (2013, Stata statistical software: StataCorp, College Station, TX, USA).

## 3. Results

There were 172 eligible patients for the study with a 32.56% response rate: 56 patients were surveyed, 38 male, 18 female. There were no differences in age, gender and type of surgery between the surveyed group and the eligible group. The mean age of the children at the surgery was 2.9 ± 4.5 years, with a minimum of 0 and a maximum of 17.2 years, while the mean age of the children at the time of the interview was 4.4 ± 6.5 years with a minimum of 1 year and a maximum of 17.6 years. All patients underwent a thoracotomy to resolve their disease and they were divided into three groups based on their disease (Table 1). We used three different anesthetic strategies: (1) general anesthesia with sevoflurane, vecuronium bromide and fentanyl (36 pts equal to 64.2%); (2) general anesthesia and thoracic epidural (10 pts equal to 17. 9%), (3) general anesthesia and intercostal nerve block (10 cases equal to 17.9%).

Postoperative pain therapy was performed mostly with intravenous morphine at a dose of 0.01 mg/kg/h or a continuous epidural with 0.125% ropivacaine at 0.1 mg/kg/h (Table 2).

The presence of postoperative pain and the efficacy of the administered therapy assessed using the hospital protocol are reported in Table 3. No patient presented pain before surgery. In the seven days following the operation, discharge and telephone interview, pain and Pain Management Index (PMI) were assessed (Table 4). It shows that 21 patients (37.5%) out of the children studied presented at least one painful episode in the immediate postoperative period: 12 (21.4%) on day 1, 10 (17.8%) on day 2, 8 (14.2%) on day 2, 5 (9%) on day 3 and only one had pain on discharge. NIPS and COMFORT scales were used for none of these patients. Of these 21 patients, 77% (17 patients equal to 30% of the total) presented at least one episode of moderate-severe pain, while 4 (17.4%) presented episodes of mild pain. The calculation of the PMI carried out in correspondence with pain showed a median value of −1 (SD ± 1 with a minimum of −2 and a maximum of +3) and it reveals an insufficient pain therapy. At the time of the interview, 3 children (5.3%) showed moderate chronic neuropathic (burning) pain on the surgical scar and they were from a minimum of 1.5 years to a maximum of 10.2 years old, with a mean of 4 ± 3.6 years. At the time of surgery, these children were aged between a minimum of 6 months and a maximum of 3 years, with a mean of 1.5 ± 1 year. Only one of them have had one moderate pain episode during the immediate postoperative week that was treated adequately (PMI = 0), while the others have not had pain during the postoperative period. At the time of the interview, all the children were taking paracetamol to relieve pain (PMI = −1).

## 4. Discussion

Chronic postsurgical pediatric pain literature is sparse with many gaps on its precise pathophysiology [15,16].

In particular, the causes of post-thoracotomy pain, like chronic pain after surgery, are not well known. Female gender, psychological factors, genetic factors and nerve injury during surgery appear to play an important role in pre-and post-thoracotomy pain [1,7,17,18].

Few papers describe the incidence of acute postoperative pain in children. It can vary from 23% described by the Swedish authors to 70% on the 1st day, to 64% on the second day, with a progressive improvement up to 28% one week after the intervention described by American authors [19,20]. However, no paper describes the incidence of acute post-thoracotomy pain in children, so we can only compare our data with those on acute postoperative pain related to other kinds of surgery. Despite the lack of use of devices of proven efficacy in pediatrics such as the patient controlled analgesia (PCA) and the lack of multimodal pain therapy, the incidence of acute post-thoracotomy pain in our series is not high [8,21]. There was no statistically significant difference between the type of anesthesia and the incidence of post-operative pain during the first postoperative week (Table 5). The latter finding contrast with what has been indicated by previous studies. Batoz et al., for instance, found that high pain scores in the first 24 postoperative hours were associated with an augmented risk of CPTPS or chronic pain following orthopedic surgery [22].

Like Chou et al. [8], we found a high incidence of pain during the first week after surgery (37.5%). Nevertheless, at discharge, only one of them presented pain with the need for a home prescription of analgesic therapy and only three had chronic post-thoracotomy pain at the interview. In fact, neuronal plasticity appears to be increased in children and this would allow their nervous system to recover earlier and better than adults after injury [23]. Chronic pain is mainly due to central sensitization and reduction of inhibitory pathways and modified gene processing in the central nervous system [19,24,25,26]. The mechanisms of persistent postsurgical pain in the pediatric population should be better explained. Probably, biological, psychological and socioenvironmental factors are involved, although the weight of the single components is difficult to establish [27].

In our study, the incidence of chronic post-thoracotomy pain we found is 5.3% (3 patients out of 56), a value of uncertain interpretation because there are few papers on this subject in pediatric age. These findings are positioned between 16% of 88 adult patients who underwent thoracotomy in childhood or youth as reported by Kristensen et al. [7] and according to the data of Chou et al. [8] who found only 1 (1.94%) patient out of 51 children operated for non-elective thoracotomy. In our series, all three of our children with chronic pain had moderate neuropathic (burning) pain in the surgical scar, as described by other authors for both chronic post-thoracotomy pain and chronic post-surgical pain [7,22,28,29,30].

## 5. Study Limitations

This study has several limitations. The main limitation is that the sample appears to be too small to draw firm conclusions. With a larger sample, the margin of error of the estimate would be reduced, for example by tripling the sample the error of the estimate is reduced to 7.6%. Consequently, with a tripled sample of 168 patients, maintaining the same proportions of pain obtained in the sample equal to 56, the *p*-value for the chi-square test would be 0.05. Furthermore, the papers that investigate this particular type of pain in pediatric age are very few. Moreover, chronic pain is a very difficult field of research as the patient’s interview is almost always conducted by telephone and not in person. This fact jeopardizes the response of many patients, especially in the pediatric field, where the possible reasons can be many and of different nature. For example, reasons that can lead to non-response can be change of address, change of telephone number, conflict within the parental couple, death of the patient, disputes with the hospital, language barrier and technological barriers. Another reason is that in the pediatric field the case history is generally lower than that of adults. In addition, in this study we analyzed only non-oncological pathologies, precisely to avoid bias regarding oncological therapies and this further narrowed the search field.

In general, with regard to chronic pain in the pediatric field the request for micro-trials is becoming increasingly pressing, as the publication rate is low and first lines of evidence are needed to treat patients [31]. Consequently, there are few publications on CPTPS in pediatric age. Our study meets the new definition of chronic postoperative pain from the International Association for the Study of Pain (IASP) [32,33]. Furthermore, it confirms the low incidence of CPTPS in pediatric age.

Apart the limited number of patients recruited, another limitation is the retrospective model and. Nevertheless, the paper is just a snapshot of our patients’ chronic pain because all the interviews were carried out at the same time and, therefore, at various intervals of time from each surgery.

## 6. Conclusions

Despite its limitations, this study confirms the low incidence of chronic post-thoracotomy pain syndrome in children. Probably multi-year and multi-center prospective studies involving a larger number of patients are needed for obtaining more accurate data on the extent of the issue. Finally, other research is warranted to dissect the precise mechanism of postoperative chronic pain in the pediatric population.

## Figures and Tables

**Table 1:** Disease division groups.

Group	Number	%	Pathology	Number
A	47	83.9	Pul.Seq.	21
			CCAM	14
			L.Emph.	7
			Pul.Cyst.	4
			TE. Fistula	1
B	7	12.5	Pectus	7
C	2	3.6	Diaph. Relax.	1
			Int. Ang.	1

Group A = Congenital Pathology of the Lung; Group B = Congenital pathologies of the thoracic cage, Group C = degenerative pathologies. Pul.Seq. = Pulmonary Sequestration; CCAM = Congenital Cystic Adenomatoid Malformation; L.Emph. = Lobar Emphysema; Pul.Cyst. = Pulmonary Cyst; TE. Fistula = Thracheoesophageal fistula; Pectus = Pectus Excavatum; Diaph.Relax. = Diaphragmatic Relaxatio; Int.Ang. = Intestinal Angiomatosis.

**Table 2:** Postoperative Analgesia.

Postoperative Analgesia	Frequency	%
Morphine iv continuous inf	41	73.2
Continuous thoracic epidural	12	21.4
Tramadol	2	3.6
Ibuprofen	1	1.8

Abbreviations: iv, intravenous; Inf, infusion.

**Table 3:** Pain Assessment Scales used.

Pain Scale	Frequency	%
FLACC	39	69.6
NRS	12	21.4
COMFORT	2	3.6
CRIES	2	3.6
NIPS	1	1.8

**Table 4:** Trajectories of pain and PMI.

					Pain after Surgery													
Demographics-Clinics					Day 0		Day 1		Day 2		Day 3		Day 4–7		Dismission		Interview	
Pathol	SurAge	IntAge	Anesth.	PTher	A.S.	PMI	A.S.	PMI	A.S.	PMI	A.S.	PMI	A.S.	PMI	A.S.	PMI	A.S.	PMI
Pul.Seq.^F^	7 y 2 m	7 y 10 m	GA	Mor ci	4	0												
CCAM^M^	1 d	1 y	GA	Mor ci	2	−1												
Int.Ang.^F^	6 m	1 y 7 m	GAInt	ThorEp			5	−1	5	−1			4	−2				
L.Emph.^F^	15 y 4 m	17 y 6 m	GAInt	Ibupr	6	−1	2	−1	2	−1	3	−1	5	−2				
CCAM^F^	1 y 9 m	2 y 9 m	GA	Mor ci	2	0												
L.Emph.^M^	3 y 9 m	7A	GAT7-8	ThorEp	7	+2							6	+3				
Pul.Seq.^M^	6 m	3 y 6 m	GA	Mor ci	6	−1												
Pul.Cyst.^M^	3 y	4 y	GA	Mor ci	4	−2	5	−1	5	−1	5	−1	4	0				
CCAM^M^	7 m	2 y 7 m	GAInt	Mor ci	3	−1												
Pul.Seq.^M^	2 y 9 m	5 y 1 m	GA	Mor ci									4	0				
CCAM^M^	1 y 10 m	8 y 7 m	GAInt	Mor ci	3	−1	6	+1										
CCAM ^F^	7 m	3 y 4 m	GAInt	Mor ci	4	1												
Pul.Seq.^F^	1 y 7 m	3 y 3 m	GAT9-10	ThorEp	3	1	5	0	4	0								
Pectus^M^	8 y	11 y	GA	Mor ci							5	−1						
Pectus^M^	14 y	17 y	GA	Mor ci					6	−1								
Pectus^M^	14 y 6 m	17 y 6 m	GA	Mor ci									5	−1				
Pectus^F^	12 y	13 y	GA	Mor ci			3	−1	3	−1								
Pectus^M^	14 y 6 m	15 y 3 m	GA	Mor ci			4	−1	4	−1	2	0	4	−1				
Pectus^M^	17 y 3 m	17 y 4 m	GA	Mor ci	4	−1	4	−1			4	−1	4	−1	4	−1		
Pectus^M^	10 y 8 m	11 y 7 m	GA	Mor ci			4	+2	4	+2			4	−1				
CCAM^F^	1 y 7 m	7 y 9 m	GA	Mor ci			4	0									4	−1
Pul.Seq.^M^	6 m	18 m	GAT7-8	ThorEp													5	−1
Pul.Cyst.^M^	3 y	4 y	GA	Mor ci													4	−1

Legend: Pathol = Pathology; SurAge = Surgical Age; IntAge = Interview Age; Anesth = Anesthesia; PTher = Postopewrative Pain Therapy; Pul.Seq. = Pulmonary Sequestration; CCAM = Congenital Cystic Adenomatoid Malformation; L.Emph. = Lobar Emphysema Pul.Cyst. = Pulmonary Cyst; TE. Fistula = Thracheoesophageal fistula; Pectus = Pectus Excavatum; Diaph.Relax. = Diaphragmatic Relaxatio; Int.Ang. = Intestinal Angiomatosis; GA = General Anesthesia; GAInt = General Anesthesia+ Intercostal Block; GAT^(level)^ = General Anestesia + Thoracic Epidural; Mor ci = Morphine continuous infusion; ThorEp = Torachic Epidural; Ibupr = Ibuprofene ; F = Female; M = Male; d = day; y = year; m = month.

**Table 5:** Postoperative pain and anesthesia.

Group	N	Patients with Postop-Pain (%)
A	36	14(38.9)
B	10	2(20.0)
C	10	5(50.0)
Total	56	21(37.5)

Group A: General Anesthesia. Group B: General Anesthesia + Thoracic Epidural. Group C: General Anesthesia + Intercostal Block. Chi square statistic is 2.0030, the *p* value is 0.442.

## Data Availability

The data can be consulted from the corresponding author for a reasonable reason.
